# Understanding the barriers and facilitators of COVID-19 risk mitigation strategy adoption and COVID-19 vaccination in refugee settlements in Uganda: a qualitative study

**DOI:** 10.1186/s12889-023-16320-4

**Published:** 2023-07-20

**Authors:** Robin E. Klabbers, Timothy R. Muwonge, Scovia Ajidiru, Sukanya Borthakur, Andrew Mujugira, Monisha Sharma, Patrick Vinck, Phuong Pham, Connie Celum, Rosalind Parkes-Ratanshi, Kelli N. O’Laughlin

**Affiliations:** 1 Department of Emergency Medicine, University of Washington, Seattle, WA USA; 2 Department of Global Health, University of Washington, Seattle, WA USA; 3 Infectious Diseases Institute, Makerere University, Kampala, Uganda; 4 Medical Teams International, Kampala, Uganda; 5 Harvard Humanitarian Initiative, Harvard University, Cambridge, MA USA; 6 Department of Epidemiology, University of Washington, Seattle, WA USA; 7 Department of Medicine, University of Washington, Seattle, WA USA; 8 Department of Psychiatry, University of Cambridge, Cambridge, UK

**Keywords:** COVID-19, Refugee, Vaccination, Risk mitigation, Uganda

## Abstract

**Background:**

Perspectives on COVID-19 risk and the willingness and ability of persons living in refugee settlements to adopt COVID-19 prevention strategies have not been rigorously evaluated. The realities of living conditions in Ugandan refugee settlements may limit the extent to which refugees can uptake strategies to mitigate COVID-19 risk.

**Methods:**

In-depth qualitative interviews were conducted between April 2021 and April 2022 to assess COVID-19 knowledge, risk perception, prevention strategy adoption including COVID-19 vaccination, and COVID-19 impact on living conditions in refugee settlements in Uganda. Interview participants included 28 purposively selected refugees who called into “Dial-COVID”, a free telephone COVID-19 information collection and dissemination platform that was advertised in refugee settlements by community health workers. Interviews were analyzed using a combination of deductive and inductive content analysis. Emerging themes were mapped onto the Theoretical Domains Framework to identify domains influencing prevention behavior. Results were synthesized to provide intervention and policy recommendations for risk mitigation in refugee settlements for COVID-19 and future infectious disease outbreaks.

**Results:**

The COVID-19 pandemic detrimentally impacted economic and food security as well as social interactions in refugee settlements. Youth were considered especially impacted, and participants reported incidents of child marriage and teenage pregnancy following school closures. Participants displayed general knowledge of COVID-19 and expressed willingness to protect themselves and others from contracting COVID-19. Risk mitigation strategy uptake including COVID-19 vaccination was influenced by COVID-19 knowledge, emotions surrounding COVID-19, the environmental context and resources, personal goals, beliefs about the consequences of (non)adoption, social influences, and behavior reinforcement. Resource constraints, housing conditions, and competing survival needs challenged the adoption of prevention strategies and compliance decreased over time.

**Conclusions:**

Contextual challenges impact the feasibility of COVID-19 risk mitigation strategy uptake in refugee settlements. Pre-existing hardships in this setting were amplified by the COVID-19 pandemic and related lockdowns. Targeted dispelling of myths, alignment of information across communication mediums, supporting survival needs and leveraging of respected role models are strategies that may hold potential to mitigate risk of infectious diseases in this setting.

**Registration details:**

World Pandemic Research Network – 490,652.

**Supplementary Information:**

The online version contains supplementary material available at 10.1186/s12889-023-16320-4.

## Background

During the height of the COVID-19 public health emergency, the World Health Organization recommended physical distancing, face mask wearing, regular hand hygiene, avoiding crowds, COVID-19 vaccination, and self-isolation as effective personal protective measures to prevent COVID-19 transmission and attenuate disease severity [1]. The extent to which individuals were willing and able to adhere to these mitigation strategies, however, was likely context dependent.

In refugee settlements in sub-Saharan Africa where individuals often live in high-density housing and access to clean water and sanitation is not guaranteed, social distancing and regular hand hygiene may not have been feasible [2, 3]. Meeting basic survival needs such as obtaining food and firewood likely necessitated that persons leave their home, posing a challenge to measures like quarantining or isolating at home [4]. The relatively low caseload observed in refugee settlements in the first two years of the pandemic, may also have impacted how COVID-19 risk was perceived in these settings and consequently, the willingness of refugee populations to adopt preventive strategies [5]. Finally, limited access to information resulting from varying literacy levels and language diversity, as well as historical incidents of medical abuse of vulnerable populations may have caused refugees to distrust recommendations [6].

Few studies have taken an in-depth approach to understand the possibilities and impossibilities of COVID-19 risk mitigation from the perspective of refugees living in refugee settlements in sub-Saharan Africa [3, 7]. Population surveys tracking COVID-19 risk perception and the adoption of preventive strategies have generally not included refugees, asylum seekers, or internally displaced populations and only a handful of surveys have captured specific behavior including COVID-19 vaccination acceptance among these populations [8, 9].

Uganda has 1.5 million refugees and is ranked the third largest refugee hosting nation in the world [10]. 94% of refugees in Uganda live in refugee settlements. These refugee settlements are often located in border regions and refugee movement patterns to and from neighboring countries including the Democratic Republic of the Congo (DRC) and South Sudan add a layer of complexity to outbreak surveillance and response as described in the context of the Ebola outbreak in 2018 [11].

The first case of COVID-19 was confirmed in Uganda on March 21st, 2020, and the country experienced the first, second, and third wave of the COVID-19 pandemic from August 2020 – January 2021, May – August 2021, and December 2021 – January 2022, respectively [12, 13]. Two national lockdowns were imposed from March 18th, 2020 – May 26th, 2020, and from June 7th, 2021 – August 2nd, 2021, during which schools, public transportation, and formal workplaces (except for essential services) were closed, and inter-district travel was suspended [14]. As part of the COVID-19 response for refugee populations, the United Nations High Commissioner for Refugees (UNHCR) and the Office of the Prime Minister (OPM) worked together with implementing partners to train healthcare personnel, strengthen surveillance and infection prevention and control, manage quarantine facilities, and perform contact tracing. In March 2021, Uganda received its first COVID-19 vaccines and in May 2021, the first vaccination campaigns were initiated in refugee settlements [15, 16]. While Ugandan nationals and refugees were treated equally, vaccination levels among refugees were almost ten-fold lower than among nationals in November 2021 (0.86% versus 8.2% respectively had received a first COVID-19 vaccine dose) [17]. After intensive social mobilization, engagement, and mass vaccination campaigns led by the Ministry of Health in partnership with the Infectious Diseases Institute and other partners in five districts in West Nile, vaccination uptake among refugees increased to 33.3% for the first COVID-19 vaccine dose and 10.6% for the second COVID-19 vaccine dose in October 2022 (an uptake only slightly lower than national uptake in at the time) [18, 19].

Conducted one year into the COVID-19 pandemic, the objective of this study was to understand the extent to which refugees living in Ugandan refugee settlements were willing and able to adopt prevention and control measures and identify barriers and facilitators to risk mitigation strategies in this context with the ultimate goal of formulating recommendations for interventions to optimize risk mitigation for COVID-19 moving forwards and for other infectious disease outbreaks in humanitarian contexts in the future. In addition, we aimed to assess the impact of COVID-19 on living conditions in this setting.

## Materials and methods

### Study setting

Most refugees in Uganda live in refugee settlements located in the northern (57%), southwestern (32%) and central region (4%) of Uganda and 8% live in the capital city Kampala [20]. Refugees from South Sudan and the DRC make up the largest refugee subpopulations, with 943,991 (61%) and 449,863 (29%) individuals respectively. Refugees from Burundi (3%), Somalia (3%), Rwanda (2%), Eritrea (1%), Ethiopia (< 1%), and Sudan (< 1%) make up the other subpopulations [20].

### Study design and participant eligibility

This qualitative study was conducted as part of “Dial-COVID”, a study leveraging interactive voice response (IVR) technology to understand and mitigate COVID-19 risk in refugee settlements in Uganda, the details of which are published elsewhere [21]. Briefly, as part of Dial-COVID, a toll-free mobile phone symptom surveillance and information dissemination tool was advertised in refugee settlements for refugees to call into. Participants 18 years or older who called into Dial-COVID, completed the Dial-COVID symptom survey, self-reported being refugees living in a refugee settlement, and consented to being contacted for additional COVID-19-related research were considered eligible for participation in this qualitative study.

### Sample size determination and sampling approach

Empirical guidance on effective sample sizes for qualitative research suggests that for homogenous study populations, data saturation can generally be achieved in 9–17 participant interviews [22]. Considering the diversity in the sample population for this study (age range, geographic location, country of origin) as well as the study objective of obtaining a deeper understanding of complex phenomena such as perceptions of COVID-19 risk and vaccination willingness and hesitancy, we expected that a larger sample size of 20–40 interviews would be needed to reach data saturation for themes and metathemes [23]. Purposive sampling was used to recruit a diverse sample of refugee participants with respect to sex, age, refugee settlement, and country of origin using information collected as part of the Dial-COVID symptom survey. Participants were contacted through the phone number listed in the Dial-COVID call-in database and were invited to participate in a qualitative interview. Recruitment continued until data saturation was reached. Saturation was achieved after conducting 28 interviews.

### Data collection

Interviews took place in person or over the phone based on COVID-19 movement restrictions and transportation availability. Interviews were conducted in the participant’s language of choice (Arabic, English, Kakwa, Kinyarwanda, Kiswahili, Lugbara, or Runyankore) by one female research assistant (SA) trained in qualitative research and fluent in English and Lugbara. The help of an interpreter working in this capacity in the study setting was enlisted when needed. For interviews with interpreter assistance, questions were asked in English by SA and translated verbatim in real-time by the interpreter, who also translated participant answers into English. To facilitate translation, interpreters were provided with a translated copy of the interview guide. In-person interviews took place in a private space at the health center with only the research participant, the interviewer, and occasionally an interpreter present, and were conducted in accordance with Uganda COVID-19 guidelines. Informed consent, either written or verbal for phone interviews, was obtained for all participants. For participants with limited literacy, consent forms were read aloud and if signing proved challenging, thumbprints were obtained. Following informed consent, basic demographic data were collected. Interviews lasted approximately one hour and were audio-recorded with permission to facilitate transcription. Interviews were semi-structured using an interview guide that covered topics including COVID-19 knowledge and risk perception, perspectives on barriers and facilitators of risk mitigation strategy adoption, perspectives on COVID-19 vaccines and vaccination willingness/hesitancy, and COVID-19 impact. Participants were asked both about their own behavior and about that of others in the community [interview guide included as Appendix 1]. Interview guide prompts were pilot tested prior to use. All interview participants were compensated UGX 20,000 (~$5.63 or €4.97) for their time, effort, and to cover any transportation costs they may have incurred.

### Data analysis

A combination of deductive and inductive thematic content analysis was used to identify and describe distinct themes in the conducted interviews [22]. First, topics (e.g., knowledge of COVID-19, COVID-19 prevention, COVID-19 risk perception) and subtopics (e.g., signs and symptoms, transmission, diagnosis) were extracted from the interview guide and used to create a preliminary codebook structure. Then, the first five interview transcripts were read by five researchers independently (KNO, REK, RPR, SA, and TRM) and each researcher performed open manual coding with organization of codes within the preliminary codebook structure. The resulting framework was discussed among the five researchers until a consensus was reached. Subsequent interview transcripts were analyzed by the aforementioned researchers in batches of 2–5 as they became available using the agreed upon coding framework to which new codes were added as they emerged. Coding consistency was reviewed regularly, the coding framework was iteratively refined, and code meaning saturation was evaluated for themes and subthemes [24, 25]. Adaptations to the coding framework were logged.

Concurrent data collection and analysis allowed for the refining and addition of questions to the interview guide to explore emerging themes of interest. After coding all interview transcripts, data were synthesized to assess patterns and interactions between themes across the different interviews. The Theoretical Domains Framework was used as a structure on which to map the results [26, 27]. The Theoretical Domains Framework is a set of 12–14 theoretical domains that was created to simplify and improve the practical application of existing health psychological theories. It has been used to assess barriers and facilitators to health behavior adoption, and to inform the design of behavior change data collection tools and interventions [28–30]. Here, exploring the study findings through the lens of the Theoretical Domains Framework allowed us to identify the cognitive, emotional, social and environmental factors that influence the adoption or non-adoption of COVID-19 risk mitigation strategies, and systematically organize these factors to highlight opportunities for risk mitigation optimization in this setting.

## Results

**Fig. 1:**
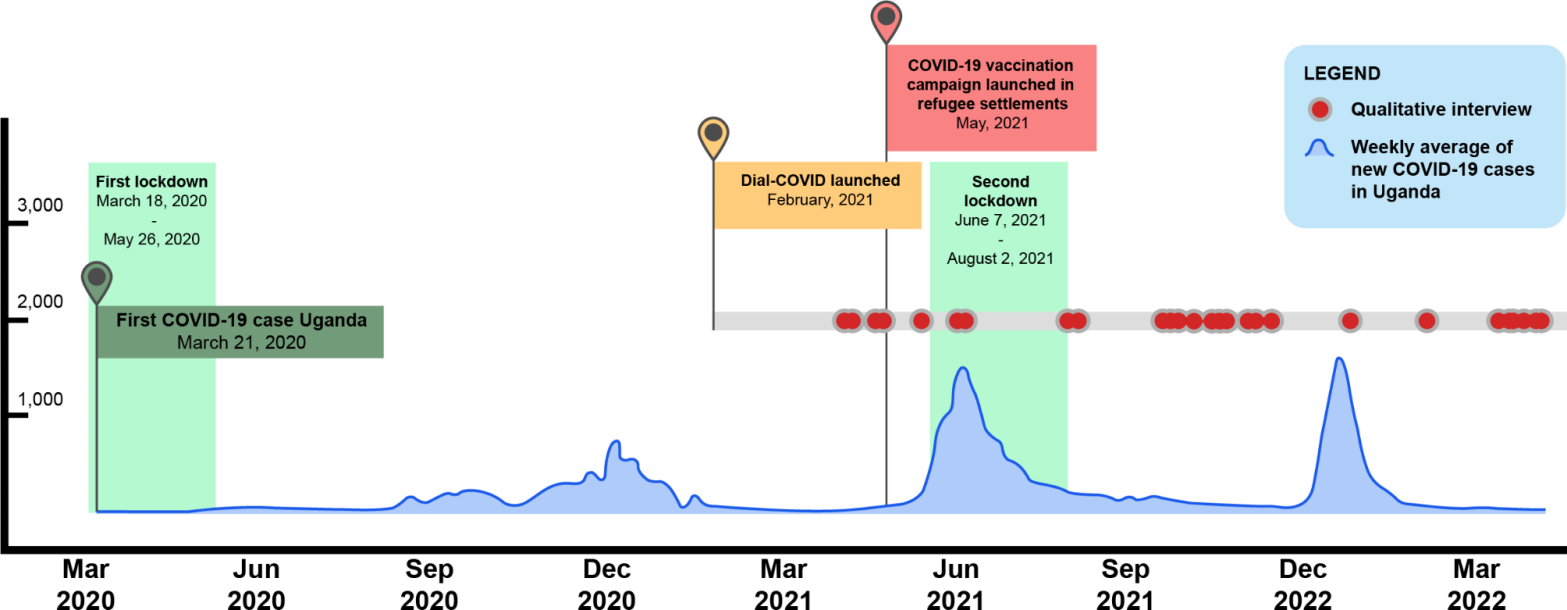
Timeline of data collection in relation to COVID-19 in Uganda

Between April 2021 and April 2022, 30 Dial-COVID call-in participants who had consented to being contacted for additional COVID-19 related research were approached for participation in a qualitative interview. One participant declined the interview due to the time burden and one participant was unable to complete the interview due to connectivity issues. A total of 28 in-depth interviews were conducted (Fig. 1). All participants were refugees and reported living in a refugee settlement; most commonly in southwestern Uganda (54%) and West Nile (42%) (Table 1). Participants were predominantly female (58%) and had a median age of 33 years (min 18, max 55 years). Education levels were low on average, with half (50%) of participants reporting to have not completed primary school education (< 7 years of schooling). Most interviews (82%) were conducted over the phone and most (86%) required an interpreter.

**Table 1:** Demographic characteristics of interview participants

		Participant total (N = 28)
**Gender**		
Female		16 (57%)
**Age (years)**		
Median [Min, Max]		32.5 [18.0, 55.0]
**Education**		
Never attended school		9 (32%)
Some primary but did not complete primary		5 (18%)
Completed primary		3 (11%)
Some secondary but did not complete secondary		6 (21%)
Completed secondary/ ordinary Level secondary school		5 (18%)
**Region**		
Central		1 (4%)
North/West Nile		13 (46%)
Southwest		14 (50%)
**Refugee settlement**		
Kyaka II		3 (11%)
Rwamwanja		11 (39%)
Palorinya		11 (39%)
Bidi Bidi		1 (4%)
Rhino Camp		1 (4%)
Kiryandongo		1 (4%)
**Country of origin**		
Democratic Republic of the Congo (DRC)		16 (57%)
South Sudan		9 (32%)
Sudan		3 (11%)
**Interview type**		
In person		5 (18%)
	Requiring an interpreter	2 (7%)
Phone		23 (82%)
	Requiring an interpreter	15 (54%)

### Health behavior related to COVID-19 risk mitigation

According to participants, most of the recommended COVID-19 risk mitigation strategies were practiced to some extent in the refugee settlement by the participants themselves and by others. Participants commonly reported protecting themselves and others from COVID-19 by handwashing, face mask wearing, and social (physical) distancing.

*“Actually for the guidelines, what I do is to always wash my hands with clean water and soap, then when in public places, wear my mask and also I give a small distance from the other person who is next to me. Those are the ones I do to prevent myself from catching the disease” (Male, age 33, South Sudan, April 2021).*

Various contextual barriers prevented other strategies such as hand sanitizing, disinfecting surfaces, quarantining or isolating at home when exposed or symptomatic, staying at home to avoid crowded places, and good nutrition from being commonly adopted. Participants noted that certain groups such as youth, were less likely to adopt risk mitigation strategies and observed in later interviews that adherence to the recommended COVID-19 prevention behaviors decreased over time as the pandemic progressed. Regarding COVID-19 vaccination, most participants either reported being willing to accept the COVID-19 vaccine when it became available (interviews conducted in 2021) or that they had already been vaccinated (interviews conducted in 2022).

### Theoretical domains Framework constructs influencing COVID-19 risk mitigation behavior

Findings from qualitative interviews were mapped onto the Theoretical Domains Framework to identify constructs driving the adoption of COVID-19 risk mitigation strategies in refugee settlements (Table 2).

**Table 2:** Interview findings mapped onto the Theoretical Domains Framework

Domain	Findings
**Knowledge**	Participants were aware of COVID-19 and knew its symptoms, how it is transmitted, how it can be diagnosed, and how it can be prevented. Participants were less knowledgeable about COVID-19 vaccines and wanted more information on this topic. Misconceptions about the COVID-19 vaccine existed.
**Emotion**	Fear of COVID-19 infectiousness and lethality motivated risk mitigation strategy adoption. A lack of knowledge regarding COVID-19 testing procedures resulted in fear of testing. Stigmatization of individuals with COVID-19 decreased over time.
**Environmental Context and Resources**	Adaptations to the physical spaces and activities in refugee settlements facilitated social distancing. Lack of supplies limited the ability to sanitize hands and disinfect surfaces. The provision of masks, supplies required for home handwashing stations and soap facilitated masking and hand washing practices. High-density housing and communal living practices made social distancing and isolating at home challenging. Basic survival needs limited the ability to stay at home to avoid crowded places and quarantine.
**Goals**	Individuals adopted COVID-19 risk mitigation strategies to protect themselves and their family from COVID-19. COVID-19 vaccination was thought to facilitate return to income generating activities.
**Beliefs about consequences**	The perception of risk of contracting severe disease associated with not following prevention measures depended on COVID-19 prevalence and beliefs surrounding COVID-19 vaccine effectiveness. Low risk perception was associated with decreased adoption of prevention measures.
**Social influences**	Most participants reported making their own decisions about whether or not to accept the COVID-19 vaccine. In some cases, participants accepted COVID-19 vaccination following encouragement from health workers or family members.
**Reinforcement**	COVID-19 prevention guidelines were reinforced formally and informally through social pressure, punitive action for non-adherence and by making adherence a requirement to access vital services.

*Based on Cane et al.[27]

#### Knowledge

Non-adoption of mitigation strategies by interview participants was generally not the result of a lack of knowledge about COVID-19. All participants had heard of COVID-19 and demonstrated an understanding of COVID-19 symptoms, transmission, and diagnosis. Several participants mentioned China as the origin of COVID-19 and many emphasized COVID-19’s global presence, its contagious nature, and potential to result in mortality.

*“COVID-19 is an airborne disease that can be got within a short period from an infected person. It is a very dangerous disease that kills and it has killed very many people in many places”* (Male, age 38, Sudan, April 2022).

Participants knew how to protect themselves against COVID-19 and described a clear link between contracting the disease and not adhering to COVID-19 protective measures.

*“Everyone is at risk of getting COVID for as long as you don’t follow the standard operating procedures. Especially if you are not putting on the mask, going in public places and not washing hands, and sanitizing, you will get the disease”* (Male, age 36, DRC, August 2021).

Participants described that generally people in the settlement had been educated about COVID-19, but that a minority believed they cannot get the disease. This minority believed that COVID-19 did not affect Black people and that they therefore did not need to follow the risk mitigation guidelines.

*“There are those in the village who refuse to follow the measures saying that Corona is not their disease, so there is no need to put on masks and follow other things like social distance and washing the hands…they are saying this disease is not for them but it’s for the whites, that it can’t kill them because, for them, they are strong”* (Female, age 28, DRC, June 2021).

At that point in time, June 2021, few COVID-19 cases had been diagnosed in refugee settlements. Most interview participants had only heard of COVID-19 cases elsewhere and had not personally been exposed to anyone with COVID-19.

Given that initially, the pandemic was largely taking place elsewhere, participants described in earlier interviews (April – August 2021) that they had not seen cases of COVID-19 themselves and instead relied on the information about COVID-19 that was provided to them. Sources of trustworthy COVID-19 information listed by participants included the radio, healthcare workers at the health facility, and village health teams (VHTs) that disseminated information by going door to door and through megaphone announcements at places of congregation such as food distribution sites. Other sources of information participants mentioned were television, posters, social media, church, and others in the community. Generally, participants trusted the information they received. The track record of credibility of those providing information and reporting of the same information by multiple sources supported its trustworthiness. Witnessing COVID-19 in the settlement and the restrictions implemented throughout Uganda to prevent COVID-19 transmission negated any lingering doubt among participants that COVID-19 was real.

*“I trust the information because I know if it’s not true, it would only come from one source but all these things are talking the same things about Corona so I believe it’s true information. And even because of that information, people learned how to protect themselves and now the rate of Covid has gone down”* (Female, age 18, DRC, November 2021).

*“I get that information through the radio and radio talks and sometimes on the television and sometimes some posters in the camp… I trust it because I have seen how people are suffering from this disease. And also these people of radios and the government cannot lie to its people. They always give education to people on facts so that people are saved from dying. Its good information and that is why I trust it and I am now following it”* (Male, age 36, DRC, August 2021).

Information about COVID-19 vaccines was seemingly slower than information about COVID-19 risk mitigation strategies to permeate the settlements. In earlier interviews (conducted prior to August 2021), several participants reported hearing something about the existence of a COVID-19 vaccine, but were unsure whether it was true. Knowledge regarding the vaccine was mainly limited to being able to name the high-risk groups initially eligible to receive it. A few participants could name specific COVID-19 vaccine manufacturers including the AstraZeneca and Johnson & Johnson vaccines. Gaps in knowledge among some participants including lack of awareness that vaccinated individuals have a reduced risk of hospitalization and death but may still experience COVID-19 symptoms led to distrust of vaccine effectiveness.

*“I think this vaccine does not work for sure, because even the doctors were also saying people should still put on masks after getting the vaccine. Then another thing that makes me believe this vaccine is fake and does not work is because I became sick after getting the vaccine and the signs were just like for COVID. For example, I lost appetite for food, I had too much cough and I was sneezing and also I did not feel the taste of anything and for me, I knew it was COVID. So that vaccine to me does not work”* (Female, age 35, South Sudan, February 2022).

Several interviewees reported needing more information to determine vaccine effectiveness and expressed wanting to see others vaccinated before accepting. Multiple participants had heard of blood clotting complications following the AstraZeneca vaccine and feared possible side-effects of vaccination. Participants reported rumors in the settlement that the COVID-19 vaccine led to infertility, lowered life-expectancy, was spreading COVID-19, contained satan or metals, and would allow behavior control by the countries producing the vaccine.

#### Emotion

Participants were aware of the potential severity of contracting COVID-19. Fear of COVID-19, specifically its lethality and the speed with which it spread was a strong motivator of risk mitigation strategy adoption. Participants expressed concern about their own health, the health of their families, and that of the wider community.

*“I am concerned and I wish that people should continue protecting themselves from getting COVID because a healthy community is what makes everyone happy, but when people are sick, there will be no happiness”* (Female, age 39, DRC, November 2021).

Especially in the earlier interviews, at the time when COVID-19 had not spread to refugee settlements, the unfamiliarity of the disease and the stories circulating about it contributed to participants’ fear.

Participants also mentioned fear of COVID-19 testing. Several interviewees described being afraid to test for COVID-19 because they did not know what the procedure entailed or what to expect. In most cases, this fear of the unknown did not prevent participants from testing for COVID-19 when they were symptomatic.

*“At first, I had a lot of fear because I did not know how the testing is done, I thought they were using these big syringes but when I reached, the doctor told me how it is done and told me not to worry because the process is quick and not so painful”* (Male, age 39, DRC, March 2022).

In earlier interviews, some participants described stigmatization of individuals diagnosed with COVID-19 resulting from fear of COVID-19 transmission, even after those individuals had recovered.

*“People at the village level … will run away from that person. They will isolate that person and will never want to interact with him or her or come close… The community, starting from the immediate neighborhood, children, and adults are fearing every person who comes from that family”* (Female, age 28, DRC, June 2021).

In later interviews, as awareness and understanding of COVID-19 increased, participants explained that stigmatization related to COVID-19 disappeared.

*“For us, we believe that this COVID has come to stay. Of course from last year, that is 2020 when COVID had just come in, people were alert, wearing masks and all sorts of things, but this year 2021, I see people do not have that issue of stigmatization. They take it as a normal thing and they even are saying if those with HIV/AIDS do not die there and then, what of COVID which can be treated? So, now it is just a normal circumstance here at the camp. Though last year, honestly last year, people used to fear to the extent that they run away from you, they isolate you, but right now, I see that mind has changed”* (Male, age 23, DRC, October 2021).

The availability of COVID-19 vaccines and the evolution of the COVID-19 pandemic globally changed COVID-19 risk perception in the settlement. As vaccines became available, fear of COVID-19 diminished, and participants described that adherence to recommended COVID-19 risk mitigation strategies like masking, social distancing, and staying at home when possible decreased among others in the settlement.

*“People think Corona is over and others are saying they are used to the disease. They are no longer scared like the way they were when it had just started. Also now, there is the vaccine and many people are going to get it, so the rate at which they follow the measures has drastically reduced. Others are even frustrated more by the hardships brought by COVID and they don’t care about it anymore”* (Female, age 35, South Sudan, February 2022).

#### Environmental context and resources

Available resources and the environmental context of the refugee settlement strongly impacted the ability to adopt COVID-19 risk mitigation strategies in this setting. Participants explained that when supplies like masks and the materials required for hand washing were provided to them, these measures became easy to follow.

*“Masking is not hard, because we have masks which were distributed by the UNHCR and everyone got six masks so when you put on one and it gets dirty, you remove it, wash it and then put on another one. When that one is dirty, you remove and put on another one like that”* (Male, age 46, DRC, November 2021).

*“Even all homes have a handwashing facility at the entrance, because we were all given jerrycans and soap specifically for that reason. At the food distribution point, there is a hand washing facility, even in the church and the hospital. So, washing hands is not a problem, because they gave us everything for free and they even increased the number of boreholes, so that is not a challenge at all”* (Female, age 39, DRC, November 2021).

Participants noted the importance of providing and replenishing supplies. Prior to November 2021, participants described that refugees had only been given one face mask per person which meant that when it required washing or was lost, they were unable to protect themselves. Even though most of the required supplies were available in the settlement at a small cost, the cost was often prohibitive. Individuals attempted to overcome financial barriers by using the supplies that were available to them. One woman said, “*We were told that if we don’t have sanitizer, we should put a piece of soap in water and it dissolves and after getting a piece of clothing, dip in that water with soap and then use it to clean the tables and the chairs,”* (Female, age 32, DRC, October 2021).

Living and housing conditions in the settlement made it difficult for people to physically distance or quarantine/isolate at home.

*“This one [physical distancing] is very hard for people to follow well because of the overpopulation in the camp and even at home … For example in most homes, people eat from the same source, like they use that big tray and food is served there for everyone and we eat together. So how can you do social distancing when you are using the same tray and dish for eating even when the food is not enough? The social distance is very hard given the conditions we live in. … Even when it comes to sleeping, many people sleep in one room and sometimes people pile themselves on one mattress, so it’s hard”* (Female, age 22 years, DRC, October 2021).

Since isolating in a separate room at home was not feasible for most people in the settlement, participants stated that when someone with COVID-19 symptoms was diagnosed with COVID-19, they would go to the hospital where isolation units were available.

Quarantining/isolating and staying at home to avoid crowded places was challenged further by the need to leave the home to obtain food, either from food distribution points or other source, fetch water and collect firewood. One female participant explained:

*“Though you are with that disease, you cannot stay throughout at home. There are other needs also like this food, this fetching water. These are serious challenges. Others can be minor ones like prayers, but water and food are the serious ones. Sometimes it is a food distribution and you are alone at home and so you cannot manage to stay at home”* (Female, age 30, South Sudan, March 2021).

Participants described adaptations to the facilities and processes in the refugee settlement to make the environment more conducive to risk mitigation strategy adoption. For example, efforts were made to facilitate social (physical) distancing in community spaces such as markets, churches or food distribution points.

*“In the market, they separated the stalls with ropes, even in our savings group which we do within the community, we also practice social distance because we have plastic chairs and [place] people far from each other. In churches, they limited the number of people to attend the church services so that people can social distance. Before people used to be 400, but now they only allow 100 people at once”* (Female, age 39, DRC, November 2021).

Another woman described, “*We are practicing social distancing especially at the food distribution points, they measure one meter and they put a hole, then another one meter, they put a hole to step in like that,”* (Female, age 46, DRC, November 2021).

#### Goals

Participants reported different motivations for adopting COVID-19 prevention strategies. Some interviewees adhered to the recommended measures to minimize the risk of contracting COVID-19, or to protect their families. Several participants noted willingness to accept the COVID-19 vaccine to facilitate income generating activities. One man said, *“I am willing to get vaccinated because I know with time, they will need people with the vaccination cards to access public places, and since I sell produce, I will need to have it to do my business,”* (Male, age 36, DRC, August 2021).

#### Beliefs about consequences

The perceived connection between adopting risk mitigation strategies and the likelihood of contracting COVID-19 influenced whether strategies were adopted. Most interviewees believed that the recommended measures were effective and that not adhering to them would result in a high risk of COVID-19. As the COVID-19 caseload decreased in Uganda, however, the consequences of not following the guidelines were considered less severe than the consequences of missed livelihood opportunities. Participants described in interviews after February 2022, that the belief among others in the settlement that vaccination protected people from getting ill from COVID-19 also resulted in the acceptance of higher exposure risk and behavior change. One female explained how some disregarded prevention measures after vaccination because they felt safe with the vaccine:

*“Right now, many people have ignored following these preventive measures but there are also still a few people who follow these measures. When the vaccine was brought, many people were vaccinated, and immediately after vaccination, they abandoned these things of putting on the mask and also social distance because they are now safe”* (Female, age 55, Sudan, March 2022).

#### Social influences

While many (indirect) social influences inevitably shape health behavior, when asked directly, most participants reported that they were able to decide for themselves whether or not to adopt prevention strategies such as receiving the COVID-19 vaccine. Some participants stated that their decision to get vaccinated was influenced by health workers or family members. One participant explained, “*For me, I only asked the health worker, and he told me that the vaccine is free of charge and he also said the vaccine is good for human health so I decided to get [it] to be safe*,” (Male, age 26, South Sudan, October 2021). Another participant had input from her father. She said, “*Yes, actually I did not want to get that vaccine but my father called me and told me to go and get it*,” (Female, age 35, South Sudan, February 2022).

#### Reinforcement

Policies in the settlement reinforced COVID-19 risk mitigation behavior. Access to certain services in the settlement, including food distribution and healthcare was contingent on participants wearing a mask or washing their hands. One female explained: “*[at food distribution] they don’t allow people without face masks to enter the line and two, they take people’s temperature before joining the line and also they make everyone wash their hands before getting on the line. So those are things done there,”* (Female, age 23, South Sudan, October 2021). Adherence to COVID-19 protective measures was enforced by the police who fined or jailed individuals found to be non-compliant. Social regulation also occurred formally and informally. At certain venues, individuals were tasked with monitoring and facilitating distancing behavior and in the community, individuals reminded each other to remain compliant with COVID-19 protective measures. One woman said, “*So the market is hard to make people follow these measures but in the church, people are so organized and we have the ushers who will guide people where to sit and if the church is full, they will tell others to stay outside*,” (Female, age 22 years, DRC, October 2021). This new social norm appeared to influence the behavior of community members.

*“Whenever we are going to get things like water, food, or firewood in the host community, we put on our masks and then go and get what we want, because if we don’t put on a mask, the host community will tell us to go back that we are bringing for them disease. So even going to collect firewood from the bush, we put on our masks to avoid those issues”* (Female, age 42, South Sudan, April 2022).

### The impact of COVID-19 on life in the refugee settlement

In the interviews, participants were asked about how the COVID-19 pandemic and the related policies such as the national lockdowns had impacted life in the refugee settlement. Participants shared that the pandemic had been a strain on already challenging circumstances. One woman spoke of the challenging times during COVID-19 when she said:

*“So many bad things, like many people lost their jobs, like teachers and those who were selling clothes, many people don’t have money, and food. Some people even lost their loved ones. Others divorced because of hard conditions at home. For the school children, they missed two years without studying, meaning their study life has been affected. Many girls got pregnant. There is no peace in homes generally due to hardships. So this disease has caused a lot of harm to families”* (Female, age 35, South Sudan, February 2022).

Participants emphasized the economic implications of not being able to conduct their livelihood activities. Many non-governmental organizations (NGOs) that provided jobs in the settlement left during the pandemic. Markets were closed and restrictions were imposed on movement of people and goods which left refugees unable to sell their agricultural produce and commercial products. Loss of income led to exacerbation of poverty and reduced food distribution rations resulted in many families experiencing food insecurity. Multiple participants highlighted the negative implications of the pandemic for youth including how school closures and the associated idleness, delinquency, and lack of adult supervision resulted in child marriage and teenage pregnancy. One female told of the challenges for youth saying:

*“As Corona came in, the youths, especially the school-going ones, were kept at home due to lockdown, and most of these children … were impregnated. Others got married at an early age even when they don’t know how to manage family issues.... Other girls who got pregnant ended up aborting due to the fear of their parents … Now with others like the boys, they have become very stubborn, they take alcohol, others disrespect their parents and others have refused to go back to school even after the schools have been opened. They say they are now big and they don’t want anyone to advise them”* (Female, age 42, South Sudan, April 2022).

Participants explained that in the refugee settlements, social cohesion was of paramount importance for daily survival. Thus, social tensions caused by fear of COVID-19 transmission disrupted social ties and contributed to daily hardships. Whereas community members previously shared resources and supported one another, they now maintained their distance and interacted only with immediate family.

*“It was not only food; we used to go to look for money, we used to do barter trade, we used to hire land in the host community, and grow our own food … but all these stopped as the host communities began to fear us, the refugees, and also we feared them. So, life became hard* (Female, age 32, DRC, October 2021).

The disruption in the social fabric eased and social interactions improved after the COVID-19 vaccine became available and restrictions on movement were lifted.

While the impact of COVID-19 was overwhelmingly perceived to be negative by participants, a minority described positive consequences of the imposed prevention measures. One woman noted the improved hygiene saying, “*COVID came with a lot of changes. … on a positive side, the hygiene in the camp improved greatly, people started washing hands, cleaning around the home areas among other things,”* (Female, age 55, Sudan, March 2022). Another spoke of the positive health benefits of mask wearing when she said, “*Now on the positive side, I have realized that even in a situation when COVID is not there, putting on the mask is a very good thing because where we travel, there is a lot of dust, many other diseases,”* (Female, age 28, DRC, June 2021).

## Discussion

Our qualitative analysis found that the willingness and ability of refugees living in refugee settlements in Uganda to protect themselves against COVID-19 by adopting risk mitigation strategies between April 2021 and April 2022 of the global COVID-19 pandemic was influenced by COVID-19 knowledge, emotions surrounding COVID-19, the environmental context and resources, personal goals, beliefs about the consequences of the (non)adoption of mitigation strategies, social influences, and behavior reinforcement. Participants demonstrated general COVID-19 knowledge and expressed a desire to protect themselves and their families from contracting COVID-19. Perspectives on COVID-19 vaccination were generally favorable and most participants were willing to be vaccinated. Participants reported that COVID-19 risk perception and adoption of risk mitigation strategies in refugee settlements decreased over time as vaccines became available. The impact of the COVID-19 pandemic and the policies instated to address it in Ugandan refugee settlements was far-reaching and income generation, food security, and social interactions were negatively affected. The lives of youth in particular were disrupted, with reported incidents of child marriage and teenage pregnancy following school closures.

Our results are largely consistent with other studies conducted in Ugandan refugee settlements during the COVID-19 pandemic. Other investigators have also found that refugees in Ugandan refugee settlements generally have confidence in the ability of prevention strategies like hand-washing, facemask wearing, staying at home, and physical distancing to prevent COVID-19 transmission [31]. Investigators in the REFLECT study, a mixed-methods study conducted in thirteen Ugandan refugee settlements between May 2020 and June 2021, similarly found that refugees were generally knowledgeable about COVID-19, but that knowledge gaps existed in specific areas and myths and negative perceptions prevailed among a minority of refugees [32]. Similar to our findings, an inter-agency effort to track rumors among affected communities in Uganda found that rumours about serious health risks resulting from COVID-19 vaccination and conspiracy theories were circulating among refugee populations [33]. Despite circulating myths, 78% of refugees in Bidi Bidi refugee settlement, the largest refugee settlement in Uganda, were willing to accept COVID-19 vaccination between March and April 2021, consistent with perspectives related to vaccine acceptance shared by our interview participants [34].

Some of our results on the other hand, diverge from findings described in previous studies. In contrast to our findings, the REFLECT study found low compliance with certain preventive measures including social distancing, facemask use, use of sanitizers and disinfectants, and healthy nutritional habits among refugee populations with investigators listing competing survival needs and structural factors in the refugee settlement context such as crowded communal spaces as key barriers [35]. High adoption of risk mitigation strategies found in our study, which was conducted later in the pandemic (April 2021 – April 2022 vs. August – November 2020) when exposure to COVID-19 in refugee settlements had increased and the COVID-19 response by UNHCR and implementing partners had been scaled up in refugee settlements, suggests, that risk perception and access to materials required for the adoption of risk mitigation strategies are key factors driving health behavior in this context.

It is clear from our findings and those of others conducting research in refugee settlements in Uganda that the COVID-19 pandemic severely impacted the lives of refugees. While COVID-19 incidence in refugee settlements remained lower than among national populations, the public health measures instated to curb COVID-19 transmission had disproportionate consequences for the lives of refugees in Uganda [36]. Refugees were economically marginalized by the national lockdowns and COVID-19 prevention measures which limited access to the places where they normally conducted their business. In combination with pandemic-related scaling back of humanitarian programs this resulted in loss of income for the refugee population [37, 38]. Data from the Uganda Refugee High-Frequency Phone Survey collected between October 2020 and May 2021 show that refugees experienced more economic shocks and were less able to recover employment than Ugandans, emphasizing the disproportionate impact of the pandemic for this population [39]. A 30% cut in food relief announced by the World Food Program in April 2020 following a funding shortfall resulted in a ration reduction from 12 kg to 8 kg per refugee per month. This was followed by further ration reductions in 2022 as a result of growing global humanitarian need and increases in global food prices resulting from Russia’s invasion of Ukraine. These ration reductions are thought to have largely contributed to the heightened food insecurity experienced in Ugandan refugee settlements during the pandemic [31, 40, 41]. A strong association was found between food insecurity during the pandemic and depression among displaced and refugee adolescent youth in Kampala demonstrating the pandemic’s far-reaching impact [42]. In focus groups and qualitative interviews, refugee adolescents and youth in Bidi Bidi refugee settlement described how resource constraints and ‘idleness’ stemming from a lack of meaningful daily activities during the COVID-19 pandemic exacerbated inequitable gender norms and fueled interpersonal violence [43].

Our qualitative assessment of COVID-19 knowledge, risk perception, and prevention strategy adoption in refugee settlements contributes to the body of knowledge on the barriers and facilitators that drive COVID-19 risk mitigation in this setting and sheds a light on how the lives of refugees were impacted during the first two years of the COVID-19 pandemic. While COVID-19 is no longer a public health emergency and the global focus has shifted away from outbreak response, the lessons learned in this study remain relevant and can help to support efforts to prepare for future infectious disease outbreaks. Drawing on our findings, a number of recommendations can be formulated. First, one of the most effective interventions to prevent COVID-19 transmission or the spread of future infectious diseases in refugee settlements may be to ensure that food rations are brought back to pre-pandemic levels or are even increased. Many of the barriers to risk mitigation strategy adoption stemmed from competing survival needs. Improving food security would enable refugees to stay at home and avoid crowded placed. Second, even when disease knowledge is generally high, it is important to monitor for myths and misinformation that may be circulating among population subgroups so that targeted information campaigns can be deployed. To improve perceived credibility of information, coordination of messaging between different information sources is important. Third, there may be a role for peer champions, local celebrities, and community leaders to model desired behavior and help inspire confidence in mitigation measure safety and effectiveness through endorsement. Finally, pre-existing vulnerability is likely to be heightened during public health emergencies and special attention should be paid to vulnerable population subgroups when designing response policies.

Our study has several limitations. While interview participants were purposefully sampled to be diverse, not all population subgroups or all refugee settlements were represented. Additionally, the perspectives and lived experiences of interviewed individuals may not be representative of their demographic groups. Participants in this study were recruited from the Dial-COVID database; the knowledge and health behavior of these individuals who pro-actively called into this COVID-19 information dissemination tool may differ from that of the general refugee population. Due to national restrictions on movement and the large distances between refugee settlements, most interviews were conducted over the phone which may have impacted the interaction between the interviewer and interview participants and limited interviewees’ willingness to open up about their perceptions and experiences. Additionally, as with all research conducted with the assistance of interpreters, it is possible that nuance was lost, or meaning was changed in translation [44]. We attempted to minimize the influence of interpretation by enlisting the services of interpreters who were already working in the study setting, and were therefore familiar with the local context, customs and culture. Lastly, interviews conducted earlier and later into the study were compared and contrasted as a proxy for changes in COVID-19 perception and risk mitigation strategy adoption over time. Conducting longitudinal interviews with the same participants at different stages of the COVID-19 pandemic would have more accurately captured this evolution, but fell outside the scope of this study. The dynamic nature of the pandemic and evolution of perspectives over time as a result of greater availability of information, changing guidelines and the development of vaccines made it challenging to assess true data saturation. Interviews were not spaced uniformly across the study period and perspectives and practices during certain phases of the COVID-19 pandemic in Uganda, such as the second national lockdown during which fewer interviews were conducted, may be less well represented.

## Conclusion

The COVID-19 pandemic and national measures implemented in Uganda to prevent COVID-19 transmission worsened economic hardship and food insecurity in refugee settlements and negatively impacted social interactions. Refugees had general knowledge of COVID-19 and the importance of adopting preventive measures was well-recognized. Contextual factors including competing survival needs, housing conditions, and limited financial resources however limited their ability to adopt risk mitigation strategies. Risk mitigation in refugee settlements for COVID-19 and similar future disease outbreaks can be strengthened by providing support to meet basic survival needs, launching targeted information campaigns to dispel circulating myths, aligning messages on different communication platforms, leveraging respected community members to model behavior, and focusing on subpopulations with pre-existing vulnerability.

## Electronic supplementary material

Below is the link to the electronic supplementary material.

Supplementary Material 1

## Data Availability

The interview recordings and transcripts generated and analyzed as part of the current study are not publicly available due the sensitive nature of some of the topics covered and potential for identification of the participants, but will be made available by the corresponding author on reasonable request.

## Abbreviations

COVID-19: Coronavirus disease 2019
DRC: Democratic Republic of the Congo
FCDO: United Kingdom Foreign, Commonwealth and Development Office
IVR: Interactive Voice Response
NGO: Non-Governmental Organization
NIHR: United Kingdom National Institute for Health Research
OPM: Office of the Prime Minister
R2HC: Research for Health in Humanitarian Crises
UNHCR: United Nations High Commissioner for Refugees
VHT: Village Health Team
