# Supplementary material for: Understanding the barriers and facilitators of COVID-19 risk mitigation strategy adoption and COVID-19 vaccination in refugee settlements in Uganda: a qualitative study
Source: BMC Public Health. 2023 Jul 20;23:1401. doi: 10.1186/s12889-023-16320-4 (PMC10360310; doi:10.1186/s12889-023-16320-4)
Supplement: Supplementary file 1 — Supplementary Material 1 [file 12889_2023_16320_MOESM1_ESM.docx]

**Appendix 1 – Dial-COVID participant qualitative interview guide**

**INTRODUCTION**

Hello, my name is _______ and I am a research assistant working with the Infectious Diseases Institute and Makerere University. I am talking to you because you participated in the Dial-COVID study and agreed to let us contact you for an interview to talk more about COVID-19. During our conversation today, we will talk about what you know about COVID-19, your feelings about COVID-19 and COVID-19 prevention strategies, and your ability to protect yourself from infection.

Before we get started today, here are a few important things to know:

- We are here to learn from you. There are no right or wrong answers to anything we will be asking. We are interested in your opinions; we are not testing your knowledge in any way.
- Please stop me from moving on to a new question if you still have something you would like to say, and feel free to come back to a topic if you think of more comments as we go on.
- What you tell me will be confidential and will not be shared with anyone you know.

Do you have any questions before we begin?

Are you still willing to participate in this qualitative interview?

Is it ok that I make an audio recording of this interview?

**BACKGROUND**

Let’s get started by you telling me a little about yourself.

- In which refugee community do you live?
- How long have you lived there?
- Who do you live with?
- How many rooms are in your home?
- Does anyone in your household participate in income generating activities? If so, which ones?

**KNOWLEDGE OF COVID-19/CORONAVIRUS**

- What have you heard about COVID- 19/coronavirus?
- Please explain to me your understanding of how COVID-19 is transmitted.
- Please tell me about the signs and symptoms of COVID-19 that you know.
- Who is at risk of getting COVID-19/coronavirus?
- Who do you think is at risk of becoming seriously ill from COVID-19?
- How do we confirm someone has COVID -19?
  - *Probe for knowledge around COVID -19 testing.*
- Please tell me about how COVID-19 is treated.
- Please tell me about the chances of recovering from COVID-19.

**COVID-19 information**

- Where or from who did you learn this information about COVID-19?
  - *How much do you trust this source?*
  - *What are other sources where you could get information about COVID-19?*
  - *Tell me about any sources of information that you do not trust.*

**COVID-19 WORKFLOW/EXPERIENCE WITH COVID-19 IN THE REFUGEE SETTLEMENT**

- What happens when someone in the settlement is suspected of having COVID-19?
  - *Where would you refer someone suspected to have COVID-19?*
- What would you do if you are experience COVID-19 symptoms yourself?
- What happens when someone is diagnosed with COVID-19 in the refugee settlement?
- Have you been tested for COVID-19?
  - *If yes, tell me why you were tested and what your experience was.*
  - *What was this process like? Where were you tested?*
  - *How long did it take to find out the results? How were the results communicated to you?*
  - *Were you given any instructions or educational information after you were tested?*
  - *Did you change your behavior (e.g., self-quarantine) while awaiting the results?*
- Do you know of anybody who has been infected by COVID-19?
  - *If yes, tell me a story of what happened.*
  - *How do you think that person got COVID-19? What happened to that person?*
- What changes have you seen in the settlement since January 2020? In what ways have these changes affected you and your household?
  - *Probe for the effects of lockdown, restricted movement among others.*

**COVID-19 RISK PERCEPTION**

- How worried are you about COVID-19?
  - *What are you worried about specifically?*
- Do you think you are at risk of getting infected with COVID-19?
  - *If yes, why?*
  - *Are there certain behaviors or activities that put you at increased risk?*
- Have you taken any steps to decrease your risk of acquiring COVID-19?
  - *If yes, what have done?*

**COVID-19 RELATED STIGMA**

- If someone from your community was diagnosed with COVID-19 how would they be treated by the community?
  - *Would the person’s household be treated differently because of the COVID-19 diagnosis?*
- If someone in your household was diagnosed with COVID-19, would you treat them differently
- When someone diagnosed with COVID-19 has been treated and returns home, how are they treated by the community?

**BARRIERS/FACILITATORS TO ADOPTION OF COVID-19 PREVENTION/CONTROL MEASURES**

- How important is COVID-19 prevention to you?
  - *Why?*
- How important is it to the people in the settlement?
  - *Why?*
- What are your thoughts on the preventive measures suggested to decrease the risk of acquiring COVID-19?
  - *Probe to have a discussion about the different recommendations, e.g. Do you feel you are able to follow this recommendation? What are some challenges or circumstances in your life that may prevent you from following this?*
    - “To project yourself from COVID-19, you should stay home as much as possible.”
    - “You should wear a face mask whenever you are outside your home.”
    - “You should only leave your home for essential reasons such as obtaining food and water.”
    - “When outside your home, stay at least six feet (2 meters) away from other people, avoid groups and public transit.”
    - “Wash your hands regularly with soap and water for 20 seconds (warm water is best if possible), especially after you've been outside your home. If this is not possible, use a hand sanitizer with 60% alcohol.”
- What are your thoughts on the recommendations for protecting your family and community in case you develop symptoms of COVID-19?
  - *Probe to have a discussion about the different recommendations, e.g. Do you feel you are able to follow this recommendation? What are some challenges or circumstances in your life that may prevent you from following this?*
    - “You should avoid contact with others unless it is essential, such as when buying food or seeking medical care.”
    - “Cover all coughs and sneezes and throw away used tissues or wash dirty handkerchiefs straight away.”
    - “Clean and disinfect surfaces in your home such as door handles each day.”
    - “You should try to stay home and away from others for at least 14 days from when your symptoms first appeared.”
    - “Eat well, drink plenty of fluids, and get a lot of rest, which can help you get better.”
- Have you used any of these strategies?
  - *Why or why not?*
- Are people in the community following these recommendations?
  - *Why or why not?*
- What are the biggest challenges you experience in following the recommendations to prevent COVID-19?
- What would make it easier for you to follow the recommendations and protect yourself against COVID-19?
- Do you have any recommendations for how the COVID-19 response in the refugee settlement could be improved?

**IMPACTS of COVID-19 ON PERSONAL LIFE**

- How has the COVID-19 affected your life? What is different now about your day-to-day living since the COVID-19 pandemic started?
- Have you experienced any difficulties as a result of COVID-19?
- Tell me some of the difficulties you have experienced due to COVID-19.
  - *How has COVID-19 affected your ability to get food for yourself and your family?*
  - *How has COVID-19 affected your ability to work and earn money?*
  - *How has COVID-19 affected your ability to access healthcare or get medications?*
  - *What are your concerns about COVID-19 impacting your family and community?*

**KNOWLEDGE/PERCEPTION OF COVID-19 VACCINE**

- Share with me about what you have heard about the COVID-19 vaccine.
  - *Where did you hear that?*
  - *How well do you trust the information you have heard?*
  - *Tell me about any experiences you or the people you know may have had with the COVID-19 vaccine.*
  - *Describe your understanding about who is eligible for the vaccine and when it will be available.*
- Share with me your thoughts about if the vaccine works to prevent COVID-19 or to prevent serious illness from COVID-19.
  - *How well do you think it prevents infection?*
  - *How well do you think it prevents serious illness from COVID-19?*
  - *How well do you think it prevents death caused by COVID-19?*
- Tell me about any concerns you have about the COVID-19 vaccine.
  - *Explain about if you think the vaccine is safe or unsafe your body and how you came to think this.*
  - *Share with me about any specific worry or side-effect of the COVID-19 vaccine that you have or that you have heard about from your friends or family.*

**WILLINGNESS TO RECEIVE VACCINE/FACTORS INFLUENCING DECISION**

- If a COVID-19 vaccine were available to you at no cost, would you choose to receive it?
  - *What would you consider when deciding whether or not to take a Covid-19 vaccine?*
  - *Who influences your decision whether to receive the vaccine or not (e.g. family and friends, community leaders, health care workers, government officials, religious leaders, celebrities, people on the radio or social media)*
  - *Why would you choose to receive or not receive the vaccine?*
  - *Are there certain characteristics of the vaccine itself that influence your decision whether or not to receive it (e.g. where it is produced, which type of vaccine it is)?*

**QUESTIONS ABOUT DIAL-COVID**

- How did you hear about Dial-COVID?
- What were your thoughts when you first heard about Dial-COVID?
- What was your motivation to call into Dial-COVID?
- Did you have any concerns about calling Dial-COVID?
- Did you experience any challenges accessing Dial-COVID?
- What are people in the settlement saying about Dial-COVID?
- Tell me about some of the reasons why people may decide not to call in?
- We are seeing that it is mainly women calling in to Dial-COVID, what do you think could be a reason for this?
- We have been seeing few calls come in from West Nile compared to the Southwest do you have any explanation as to why that is?

**CLOSING**

- What was it like for you to take an interview over the phone rather than in-person?
- Do you have any final thoughts you like to share? Is there anything else that you would want to discuss?

Thank you very much for your time.

**ADDITIONAL QUESTIONS BASED ON EMERGING THEMES**

**Community**

- How has COVID-19 changed the way that the community interacts with one another?
  - *How are people coming together or not coming together?*
  - *How are people supporting each other or not supporting each other?*

**Host community**

- How has COVID-19 impacted the interactions between the host community and the refugee community?
  - *Can you share any examples of stigma or mistrust?*

**Stigma/fear**

- How does a positive COVID diagnosis impact how someone and their family are treated by the community?
  - *In your observation, how are people treated that were diagnosed with COVID months ago (i.e., people who likely recovered already)?*
- Is there hesitancy to report for testing when someone presents with possible COVID-19 symptoms because of fear of quarantine/isolation and other consequences of a positive diagnosis?

**Youth**

- What consequences have you seen in the community of youth not being in school?
  - *Since children have not been in school, what are they doing?*
    - *Have you heard of any stories of child marriage or pregnancy that seem related to the pandemic?*
  - *How well do children adhere to the preventive measures?*

**Dial-COVID platform**

- What did you think about interacting with the Dial-COVID platform? What did you think about taking this survey in which you were asked questions about COVID-19 symptoms?
  - *Tell me about your experience.*
  - *How easy or difficult was it to answer the questions using your phone?*
  - *Had you ever done something similar where you listen to questions on your phone and answer by pressing numbers on your phone keypad?*
  - **** IMPORTANT***: For the North: few people in your region have completed this survey, why do you think that is?*

**Vaccines**

- How safe do you think the COVID-19 vaccines is? How worried are you about side-effects of vaccination?
- Who influences your decision whether or not to get the COVID-19 vaccine?

**Masks**

- How effective do you think masks are in preventing the transmission of COVID-19?
  - How does this influence mask wearing practices?

**Burial practices**

- If someone dies of COVID-19, how are they buried? How is this different from before the COVID-19 pandemic?
  - What do you think of these practices?
  - How have these changes impacted the families of these victims of COVID-19?

**Lockdown**

- Describe how life in the settlement has changed since the lockdown measures in Uganda were lifted.
